# Factors associated with 2009 pandemic influenza A (H1N1) vaccination acceptance among university students from India during the post-pandemic phase

**DOI:** 10.1186/1471-2334-11-205

**Published:** 2011-07-29

**Authors:** Padmanaban S Suresh, Venkatesh Thejaswini, Thangarasu Rajan

**Affiliations:** 1 Centre for Biomedical Research, Vellore Institute of Technology University, Vellore, India; 2 Dept of Molecular Reproduction, Development and Genetics, Indian Institute of Science, Bangalore, India; 3 Aarupadai Veedu Medical College and Hospital, Puducherry, India

**Keywords:** H1N1, Vaccination, Vaccine acceptance, Assessment

## Abstract

**Background:**

There was a low adherence to influenza A (H1N1) vaccination program among university students and health care workers during the pandemic influenza in many parts of the world. Vaccination of high risk individuals is one of the recommendations of World Health Organization during the post-pandemic period. It is not documented about the student's knowledge, attitude and willingness to accept H1N1 vaccination during the post-pandemic period. We aimed to analyze the student's knowledge, attitude and willingness to accept H1N1 vaccination during the post-pandemic period in India.

**Methods:**

Vaccine against H1N1 was made available to the students of Vellore Institute of Technology, India from September 2010. The data are based on a cross-sectional study conducted during October 2010 to January 2011 using a self-administered questionnaire with a representative sample of the student population (N = 802).

**Results:**

Of the 802 respondents, only 102/802 (12.7%) had been vaccinated and 105/802 (13%) planned to do so in the future, while 595/802 (74%) would probably or definitely not get vaccinated in the future. The highest coverage was among the female (65/102, 63.7%) and non-compliance was higher among men in the group (384/595; 64.5%) (p < 0.0001). The representation of students from school of Bio-sciences and Bio-technology among vaccinees is significantly higher than that of other schools. Majority of the study population from the three groups perceived vaccine against H1N1 as the effective preventive measure when compared to other preventive measures. 250/595 (42%) of the responders argued of not being in the risk group. The risk perception was significantly higher among female (p < 0.0001). With in the study group, 453/802 (56.4%) said that they got the information, mostly from media.

**Conclusions:**

Our study shows that the vaccination coverage among university students remains very low in the post-pandemic period and doubts about the safety and effectiveness of the vaccine are key elements in their rejection. Our results indicate a need to provide accessible information about the vaccine safety by scientific authorities and fill gaps and confusions in this regard.

## Background

With the initial outbreak in Mexico during March 2009, Influenza A, H1N1 (previously known as Swine Flu) has spread to many countries within a short period of time [1,2]. World Health Organization (WHO) declared the H1N1 influenza pandemic in June, 2010 [3]. As reported by WHO, the number of deaths resulting from pandemic H1N1 was 7,826 until 22 November 2009 [4]. The swine flu outbreak also affected India and as per the Ministry of Health and family welfare, Government of India, the total number of deaths of lab confirmed cases cumulative from May 2009 to December 2010 was 2,728 [5]. Indian Government planned to vaccinate the at risk population with pandemic vaccine as an important/mitigation strategy and efforts were made to manufacture the pandemic vaccine indigenously during the pandemic period [6]. In the meantime, Ministry of Health & FW, Government of India has also imported 1.5 million doses of vaccine such as the **PANENZA**, M/s Sanofi Pasteur, France. In the first phase, health care workers from the community health centre were targeted. Serum Institute of India in consultation with World Health Organisation (WHO) and the Ministry of Health, Government of India launched its indigenously developed intra-nasal vaccine named Nasovac in July 2010. On August 2010, WHO announced that the H1N1 influenza has moved into the post-pandemic period. WHO also strongly recommended vaccination of high-risk individuals in countries where influenza vaccines are available during the post-pandemic phase. Educational institutes can become serious out break centers because of their high social contact and group living with permeable boundaries [7]. If they become the sites of transmission, they may have a negative impact on the larger communities in which they are embedded, and in addition it is observed that student behavior is often divergent from non-student adult populations [7]. Students are special population to investigate their attitudes to accept new vaccination because they are open-minded, educated and supposedly will respond quickly to public health issues. For the above reasons, Centre for Disease Control and Prevention (CDC) published a guide for strategies to be carried out against pandemic, for institutions of higher education during 2009-2010 calender year on 22-02-2010 [8]. However, studies conducted among students and health care workers in different parts of the world reported low penetration of H1N1 vaccination during the pandemic period [3,9-11]. Assessment of behavioral changes during the influenza A (H1N1) outbreak in an Indian population showed the poor behavioral response despite having acceptable knowledge and attitude towards influenza A (H1N1) [12]. We consider that it is equally important to analyze the attitude and willingness of the students, to accept influenza A (H1N1) vaccination during the post-pandemic period to gain insights if any knowledge gained by them about the concerns and safety of the vaccine transpire in action of acceptance. The continued monitoring of vaccine uptake will enable to tackle second pandemic wave, if any country would be affected in the future. These efforts of understanding the student's perspective about the H1N1 vaccine may be useful in response planning and management strategies during an outbreak as well in the post-pandemic period. In the above context, we sought to investigate the various factors influencing the acceptance of H1N1 vaccine among the university students. The study was also done aiming to identify the factors responsible for their unwillingness to accept vaccination during the post-pandemic period.

## Methods

### Participants

The target group was university students of Vellore Institute of Technology, India wherein teaching is structured around nine different schools of study, and the university offers 15 undergraduate and 30 post graduate programmes. In 2010, the total number of students registered for different courses were 17,200 and the minimum sample size to represent this population at 95% confidence level was calculated to be 580. The study population was a convenience sample and questionnaire was distributed to the students and a total number of 1000 students participated in this study. Data were collected from 802 respondents. Students participated in this study belong to different states of India.

### Data Collection and analysis

**NASOVAC **(Influenza Vaccine (Human, Live Attenuated)) Pandemic (H1N1), live monovalent vaccine was made available to the students from September 2010. The survey was conducted from October 2010 to January 2011 using the questionnaire. Questionnaire included multiple choice and Likert scale type questions regarding levels of knowledge and attitudes toward H1N1influenza pandemic and prevention measures. It was also composed of questions regarding the importance of H1N1 vaccine and reasons for avoidance of getting vaccinated. It had questions and assessment centred around five main themes: Knowledge about pandemic (H1N1) Influenza, knowledge about the mode of transmission, knowledge about H1N1 vaccination, beliefs and attitudes about H1N1 vaccination and knowledge about prevention measures for H1N1. The study was conducted after one month of initiation of the vaccination program. Questionnaire was distributed randomly. Doubts and concerns raised by the students while filling the questionnaire were addressed promptly. The completed questionnaires were collected by the volunteer students and the health care workers of VIT. The completed questionnaire was checked and if any question was left unanswered by mistake without any intention, participants were requested to fill it. It was estimated that the average time the participants took to complete the questionnaire was nearly 20 minutes.

### Statistical analysis

The data were analyzed using PRISM GraphPad, version 4.0; GraphPad Software Inc., San Diego, CA. Percentage calculation was computed for descriptive purposes and chi-square test was done to analyze the statistical significance. p < 0.05 was considered as significant.

### Ethical considerations

The study was approved by the University Human Ethical Committee (UHEC), VIT university. All the participants were given details about the purpose of the study. They were informed that they have the right to refuse to participate in or to withdraw from the study. Oral consent was obtained after explaining the purpose through written information. Those who agreed orally only participated in the study. Anonymity and confidentiality of the data derived was maintained and given assurance to the study participants.

## Results

### Declared acceptance of vaccination by the study group

#### Gender

The response rate to our questionnaire was 80.2% (802 out of 1000 approached) because few students did not return back the questionnaire and some did not want to participate in the study. Among the 802 students answered the questionnaire, 506/802 (63%) were male and 296/802 (36.9%) were female. A total of 102/802 (12.7%) students reported to have received the H1N1 influenza vaccine from the health centre of Vellore Institute of Technology, India and elsewhere. Among the 102 students who have received vaccination, 65/102 (63.7%) were female and the remaining were men. The highest coverage for vaccination was among female (p < 0.0001). (See Table 1) It is interesting to note that the representation of males among vaccinees is lower than that of females, whereas it is higher in the group intending to vaccinate. (See Table 1).

**Table 1:** Acceptance rate of H1N1 vaccination according to gender, school of study and perceptions

Variables	Respondents in survey (n = 802)	Vaccinated (n = 102)	Intending to get vaccinated (n = 105)	Deny vaccination (n = 595)	P- value
	n (%)	n (%)	n (%)	n (%)	
**GENDER**					
Male	506 (63)	37 (36.2)	85 (80.9)	384 (64.5)	X^2 ^= 46.42
Female	296 (36.9)	65 (63.7)	20 (19)	211 (35.4)	P < 0.0001
**SCHOOL OF STUDY**					
Bio-Sciences & Biotechnology	426 (53)	68 (66.6)	65 (61.9)	293 (49.2)	X^2 ^= 14.36
Others	376 (46.8)	34 (33.3)	40 (38)	302 (50.7)	P < 0.0008
**PERCEPTIONS ABOUT H1N1 VACCINE**					
Effective	500 (62.3)	78 (76.4)	65 (61.9)	357 (60)	X^2 ^= 73.61
Low effective	125 (15.5)	10 (9.8)	20 (19)	95 (15.9)	P < 0.0001
In effective	25 (3.1)	2 (1.9)	15 (14.2)	8 (1.3)	
Don't know	152 (18.9)	12 (11.7)	5 (4.7)	135 (22.6)	
**ANTIVIRAL TREATMENT**					
Effective	450 (56.1)	58 (56.8)	25 (23.8)	367 (61.6)	X^2 ^= 115
Low effective	150 (18.7)	10 (9.8)	35 (33.3)	105 (17.6)	P < 0.0001
In effective	155 (19.2)	12 (11.7)	32 (30.4)	111 (18.6)	
Don't know	47 (5.8)	22 (21.5)	13 (12.3)	12 (2.0)	
**WEARING FACE MASK**					
Effective	600 (62.3)	68 (66.6)	88 (83.8)	444 (74.6)	X^2 ^= 197.5
Low effective	170 (15.5)	5 (4.9)	15 (14.2)	150 (25.2)	P < 0.0001
In effective	12 (3.1)	11 (10.7)	1 (0.1)	0 (0)	
Don't know	20 (18.9)	18 (17.6)	1 (0.1)	1 (0.1)	
**HAND WASHING**					
Effective	340 (42.3)	12 (11.7)	50 (47.6)	278 (46.7)	X^2 ^= 297.5
Low effective	250 (31.1)	15 (14.7)	8 (7.6)	227 (38)	P < 0.0001
In effective	100 (12.4)	11 (10.7)	20 (19)	69 (11.5)	
Don't know	112 (13.9)	64 (62.7)	27 (25.7)	21 (3.5)	
**QUARANTINE**					
Effective	200 (24.9)	77 (75.4)	28 (26.6)	95 (15.9)	X^2 ^= 244.2
Low effective	200 (24.9)	5 (4.9)	27 (25.7)	168 (28.2)	P < 0.0001
In effective	200 (24.9)	1 (0.9)	35 (33.3)	164 (27.5)	
Don't know	202 (25.1)	19 (18.6)	15 (14.2)	168 (28.2)	

### School of study

426/802 (53%) of the students were enrolled in the courses related to biotechnology and microbiology under the school of Bio-sciences and Bio-technology. Among the 102 students who have received vaccination, 68/102 (66%) were from the school of Bio-sciences and Bio-technology and the remaining were from other schools that include school of engineering (various branches) and others. The representation of participants from the school of Bio-sciences and Bio-technology were higher in the group intending to vaccinate in the future. (p < 0.0008) (Table 1)

### Perceptions

Majority of the study population representing vaccinated, group intending to vaccinate and non-vaccinated perceived vaccine against H1N1 as effective (78/102, 76.4%, 65/105, 61.9% and 357/595, 60% respectively). Similar perception was observed for wearing face mask to protect against H1N1 influenza. Most of the students from the group who were vaccinated and non-vaccinated believed that anti-viral treatment is more effective against H1N1 influenza (58/102, 56.8% and 367/595, 61.6% respectively). Very few students (12/102, 11.7%) had the opinion of hand washing being more effective as a preventive measure against H1N1 in the vaccinated group. 50/105, 47.6% and 278/595, 46.7% of the students from the group intending to vaccinate and non-vaccinated respectively believed the hand washing as an effective preventive measure against H1N1 influenza respectively. It is interesting to note that majority of the students (62.7%) from the vaccinated group did not perceive the hand washing as an effective preventive measure. Quarantine guidelines were found to be effective against H1N1 influenza by 77/102, 75.4% of the students in the vaccinated group. (See Table 1.). The responses like very effective and moderately effective were pooled and defined as effective.

### Reasons given by the study group for non-compliance with H1N1 vaccination

The reasons for their unwillingness were examined by a multiple choice question which assessed their passiveness, distrust and assessment without knowing about the H1N1 vaccine. Of the 700 out of 802 students (87.2%) who had not been vaccinated, 105 (15%) had stated that their attitude is not against the vaccination and would definitely receive soon. This group of students did not respond to the question for the reasons of not being vaccinated. Among the remaining, 595 students (384-men (64.5%), 211-female (35.4%)) said that they would never want to be vaccinated, concerns were about the safety and trust (doubt about the effectiveness) of the vaccine, belief of not being in the high risk group were top major reasons for their decision. (cited by 20.5%, 24.2% and 42% respectively) (See Table 2.). Among all the reasons, the major reported reason was a belief that they were not at risk for getting the H1N1 infection (42%) (Table 2.)

**Table 2:** Reasons for denying vaccination among students in the study group

Reason	Number of students (n)	Percentage (%)
It is not safe for me	122	20.5
I do not trust it	144	24.2
I do not belong to the risk group to have it	250	42
I do not want to be an experimental animal	44	7.3
I don't know	35	5.8
Total	595	100

### Self risk perception analysis

Next it was analyzed about their self risk perception for H1N1 influenza (See Table 3.). 182 (22.6%) students among the study group perceived the risk of being infected with H1N1 as high, 406 (50.6%) students believed the risk was moderate, 210 (26.1%) believed the risk was low and remaining 4 (0.5%) students indicated that it is unknown. There was a difference in the self risk perceptions according to gender and risk perceptions for H1N1 influenza among men is much lower when compared to female (p < 0.0001; Table 3.).

**Table 3:** Self risk perception among students in the study group

Gender	High	Moderate	Low	Unknown	Total
Men	45 (8.8%)	284 (56.1%)	175 (34.5%)	2 (0.4%)	506
Women	137(46.2%)	122 (41.2%)	35 (11.8%)	2 (0.6%)	296
Total	182 (22.6%)	406 (50.6%)	210 (26.1%)	4 (0.5%)	802

X^2 ^= 160.5; P < 0.0001

### Knowledge and attitude towards H1N1 influenza

Following self risk perceptions analysis, it was analyzed about the student's knowledge and attitude towards H1N1 influenza in the study group. Analysis was done based on Likert scale questions. The answers to these questions would examine their knowledge on general well established facts, interpretation of their knowledge and perception of knowledge. The vast majority of the participants (99%) were aware of the influenza outbreak (Table 4.). 76/102, 74.5% of the vaccinated students agreed to the seriousness of H1N1 influenza. Majority of the non-vaccinated participants were not aware of the general facts regarding the seriousness of H1N1 influenza infection and transmission mode (207/595, 34.7% 284/595, 47.7% respectively). Study population belonging to the group intending to vaccinate and also vaccinated disagreed that H1N1 vaccination should be given to only high risk groups. Most of the students from the vaccinated group believed that there is not enough information available for the common public about H1N1 influenza (70/102; 68%). Majority of the students belonging to the group intending to vaccinate and also vaccinated perceived that they were adequately informed about the H1N1 influenza (73/105; 69.5%, 77/102; 75%, respectively) (See Table 4). V- vaccinated; NV- non-vaccinated and IV- Intending to vaccinate

**Table 4:** Knowledge about and attitudes toward H1N1 influenza

		I disagree			I agree			I don't know	
	V (%)	NV (%)	IV (%)	V (%)	NV (%)	IV (%)	V (%)	NV (%)	IV (%)
1. There is an outbreak of H1N1 influenza in India and world	1 (0.9)	3 (0.5)	2 (1.9)	100(98)	592(99)	102(97)	1(0.9)	0	1(0.9)
2. H1N1 influenza is serious and may cause fatalities	26(25.4)	202(33.9)	43(41)	76(74.5)	186(31.2)	59(56)	0	207(34.7)	3(2.8)
3. Transmission of H1N1 occurs from human to human	42(41)	36(6)	34(32)	59(57.8)	275(46.2)	70(66)	1(0.9)	284(47.7)	1(0.9)
4. H1N1 influenza vaccine should be given only to high risk groups	100(98)	200(33.6)	75(71)	2(1.9)	147(24.7)	25(23)	0	248(41.6)	5(4.7)
5. Vaccination against H1N1 influenza will stop the outbreak	0	85(14.2)	5(4.7)	99(97)	295(49.5)	100(95)	3(2.9)	215(36)	0
6. I am adequately informed about the H1N1 influenza	4(4)	100(16.8)	12(11.4)	77(75)	250(42)	73(69.5)	21(20)	245(41)	20(19)
7. There is enough information available about the H1N1 influenza vaccine to the common public	70(68)	250(42)	26(24.7)	27(26.4)	214(35.9)	53(50.4)	5(4.9)	131(22)	26(24.7)
8. We need antiviral drug to treat H1N1 influenza	5(4.9)	110(18.4)	17(16.1)	60(58)	70(11.7)	64(61)	37(5)	415(22)	24(24.7)

### Student's sources of information about H1N1 influenza

The main source of information for the students about the H1N1 influenza was Media (453/802, 56.4%) followed by the internet (326/802; 40.6%) and health personnel (23/802, 2.8%).

## Discussion

It has been previously reported that there was a marked failure in the control effects of H1N1 to achieve increased coverage rates for pandemic influenza A (H1N1) vaccination among students and health care workers in different parts of the world [3,9,11]. However, we have not found any report through literature search on knowledge and attitudes of University students towards the H1N1 influenza and vaccination program among Indian population and others during the post-pandemic phase. Therefore, we conducted this study to investigate student responses to H1N1 influenza and their attitudes towards vaccination during the post-pandemic phase and provide baseline data, which might be useful in response planning and management strategies.

Vaccination program was implemented by the health centre, VIT University for the students during September 2010 during the post-pandemic period. In the present study, we examined the knowledge, attitude and factors responsible for their willingness/unwillingness to accept the vaccine during the post-pandemic period (October 2010 to January 2011). This represents a good case for study and to the best of our knowledge, this is the first study to examine during the post-pandemic period and the findings will be important to formulate regulations for vaccine uptake. It was assumed that there would be a drastic outreach and knowledge on safety and side effects of the vaccine when the study was conducted. As a first step in examining the attitude, the vaccination acceptance rate was verified after the introduction in the university. The report acceptance rate of H1N1 vaccine in the survey group university students, during the four months was 12.7%. During the course of the 2009 H1N1 pandemic, a number of studies reported low coverage rates for H1N1 vaccination among Greek medical students (8%), health care workers from Spain (16.5%), Italy (18%), Scotland (49.6%), France (36.5%) and general population from China (10.8%) [9,10,13-16]. It should be noted that the survey among Indian students was conducted at a post-pandemic period and still report a much lower uptake among university students. The acceptability of the H1N1 vaccine will depend upon the cost and also people have more concerns about the safety and effectiveness of the vaccine worldwide [17,18]. In the present study, cost would not be a blocking factor for compliance with vaccination as it was given at an affordable price to the students in the VIT University and in India. The highest coverage for vaccination was found among females (p < 0.001) during the post pandemic phase. This is in contrast to the reported gender based differences in accepting H1N1 vaccination among health care workers in France and common people in France and Israel during the pandemic phase [15,18,19]. The above studies were conducted during the pandemic phase of H1N1 influenza and the higher number of males intending to vaccinate in the future from our study group was surprising but comparable to the result observed among common people in France [19]. It is interesting to note that majority of the participants who had vaccination or intending to vaccinate were from the school of Bio-sciences and Bio-technology. This is quite similar to a study which reported a significant difference in the participant's attitude to get vaccinated or deny vaccination based on the school of their study in the pandemic phase [9]. Even the H1N1 vaccination rate was higher among the biologists when compared to health care workers in Italy during the pandemic period [9].

WHO recommends good hygiene as the preventive measure in limiting the spread of H1N1 influenza but limited evidence is available regarding the impact of wearing mask, cough etiquette and hand washing [3,20]. Hygienic behavior is affected by many factors such as the timing of outbreak, self-risk perception, responsibility for others and personal habits [3,21]. Students in the study group believed that wearing facemask followed by vaccine treatment are the most effective preventive measures. This is in contrast to the previous study reported by Akan and co investigators conducted among university students of Turkey during the pandemic period where a majority of them believed that quarantine followed by hand washing, and facemask were very effective preventive measures against H1N1 influenza [3]. It is interesting to note that the majority of the participants among all the three groups (vaccinated, denied vaccination and intending to vaccinate) believed that H1N1 vaccine and wearing facemask will protect from H1N1 influenza. Most of the students in the study group believed that hand washing was moderately effective or low effective in preventive measures. In a previous study, it was stated that hand washing or wearing masks will not be as effective in comparison to vaccination as a preventive measures against H1N1 [22]. Female students in Korea washed hands more frequently during the peak pandemic period of H1N1 influenza and perceived hand washing to be more effective [23]. These differences could result from the study population demographics, the knowledge difference and might be the period of infection.

Several causes have been proposed for the low compliance to vaccination during the pandemic period [3,10,11,17]. Passiveness, distrust about the vaccine, concerns about the safety and effectiveness, belief of being not in the risk population and assessment without knowing were top key elements in the attitude of our study group towards H1N1 vaccination. These were the major reasons reported for denial of vaccination among Greek medical students, Turkey university students, Health care workers from China and common population from France during the pandemic phase [3,10,11,19]. At the time of the study during the post-pandemic period, there was much coverage about H1N1 vaccination in the media and various other information resources in India [5]. Nevertheless, the high degree of rejection could be related to the varied subjective risk perception and high belief of the students in their ability to avoid infection during the post-pandemic period. Therefore, the vaccination was probably considered as being redundant. It should be noted that the study conducted among health care workers in public hospitals of Hongkong reported no change in the potential acceptance of vaccine at different WHO pandemic alert levels (pandemic alert phase 3 and 5) [24]. Our study further reports no improvement in the acceptance of vaccine among students during the post-pandemic phase. The main reason for refusal of Indian students was fear of side effects of the vaccine, and it would be interesting to conduct studies in different groups across different countries during the post-pandemic phase.

In the present study, the self risk perception analysis showed a gender difference significantly and also more than 50% of the study group perceived the risk as moderate. It is possible that risk perception may have changed during the post-pandemic period. However, studies conducted during the peak point of the outbreak also have shown the negative attitude of the students with low self risk perception [3]. In another study conducted in Australia, perception of susceptibility of university students significantly declined with the decline in the laboratory confirmed cases [7]. In our study, the reasons for non-compliance that were stated by the respondents were not different when compared to other studies conducted during the pandemic period [3]. The self-risk perception was higher in the females consistent with the previous study, and it is known that there exist some gender differences in the perceptions of environmental health risks [3,25,26]. A cross-sectional questionnaire survey conducted among the common public in India during July-August, 2009 had shown that the males had significantly higher knowledge of H1N1 influenza compared to females, but it was females who had the significant positive attitude response towards H1N1 influenza [12]. This is in contrast to a recent study conducted in middle and high school teachers of rural Georgia, where H1N1 vaccine acceptance was associated more with male gender [27]. Our study shows similar perception to a study conducted in Korean University students during the peak-pandemic period where female participants perceived their personal susceptibility to H1N1 influenza as higher [23]. Similar gender specific differences in the attitudes were reported in Chinese general population [16]. Female participants showed higher self-risk perception and positive attitude towards vaccination in our study, and these findings are similar in both pandemic and post-pandemic period of H1N1 influenza in many study populations at different cultural backgrounds.

Knowledge and attitudes toward a pandemic are important in vaccine acceptance, and it is interesting to note that 99% of our study group knows about the outbreak of H1N1 in India and world. The seriousness of H1N1 influenza was agreed by the majority of the participants in both the group's i.e, vaccinated and those intending to vaccinate. It should be noted that this knowledge did not reflect in their actions to accept the available vaccine, and it could be possible that they have the attitude of not to worry about the disease in the future than the short term adverse effects of vaccination if any. Analysis of their knowledge on well established facts (Question 1, 2 and 3) showed the better response in the group which was vaccinated and intending to vaccinate. Majority of the students from vaccinated, and intending to vaccinate groups disagreed that the high risk individuals would highly benefit from H1N1 vaccination. We found that many of the answers from both groups (vaccinated and intending to vaccinate) reflected similar knowledge, perception of knowledge when compared to the non-vaccinated individuals. Similar findings were observed in other studies conducted during the pandemic phase of H1N1 influenza [16,28].

The most important source of information regarding influenza pandemics was from the mass media [3,12,29,30]. In the study of Kamate and coinvestigators conducted among Indians from State of Rajasthan, the maximum number of subjects obtained information related to influenza A (H1N1) from television [12]. In our study, the major group of respondents said that they knew the information from media. At the time of our study there was much coverage about the influenza A (H1N1). Most of them were unwilling to accept H1N1 vaccination and it is apparent that public information about safety and effectiveness of the vaccine along with knowledge of the H1N1 influenza is crucial to meet the vaccination targets. It appears that students are less willing to have the vaccination during the post-pandemic period also. The reasons for unwillingness as stated by the respondents were not different from the ones given in the pandemic period when the new vaccine was launched. Our findings show that Indians require information on vaccine safety, and authorities should provide the necessary data to ensure the public confidence through media as it was the major source of information for them.

This study is unique because it was conducted among the students during the post-pandemic period to analyze their attitude difference in accepting the new vaccine. However, we realize that there are several limitations that require consideration. All the information obtained was self reported and reporting bias always exists. Although the data was collected from the heterogeneous group, we targeted students who are willing to participate and give their answers. We cannot reach all the students, and some did not return the questionnaire for varied reasons (20%). More importantly, there can be a fear factor among the students, if any ignorance of them will be pointed out in their answers. The student's opinion also can be unstable. Any unexpected event could lead to drastic change in their opinion about the vaccination.

## Conclusions

Despite some of the limitations, our study outlines the low H1N1 vaccination rates among university students, India in the post-pandemic period. The participants knew about H1N1 pandemic and had sufficient knowledge derived mostly from the media. Risk perceptions were different between male and female students during the post-pandemic period. This study identifies the factors that play a major role in their unwillingness to accept vaccination. It appears that safety and effectiveness of the vaccine are the major concerns for those who are undecided about vaccination. There is a need for accessible information about vaccine safety and public education campaigns may be effective in changing their perception patterns. Unless the safety and effectiveness of the H1N1 vaccine are informed by various scientific authorities through the media, the negative attitude and non-compliance towards H1N1 vaccination will continue irrespective of the severity of the disease and different periods (pandemic or post-pandemic). Further research is warranted for different population groups in different countries during the post-pandemic phase. Such studies would expand our understanding to make guidelines for promoting vaccine against H1N1 influenza.

## Competing interests

The authors declare that they have no competing interests.

## Authors' contributions

PSS designed the study and drafted the protocol. PSS, TR conducted the data analyses. PSS, VT and TR drafted the manuscript. All authors read and approved the final manuscript.

## Pre-publication history

The pre-publication history for this paper can be accessed here:

http://www.biomedcentral.com/1471-2334/11/205/prepub
